# Supplementary material for: Circulation of Herpesvirus and Alphatorquevirus DNA in each trimester in asymptomatic women pregnant with twins
Source: PLoS One. 2025 Nov 14;20(11):e0335200. doi: 10.1371/journal.pone.0335200 (PMC12617869; doi:10.1371/journal.pone.0335200)

**Supplement Table S1**

Oligonucleotide sequences of synthetic curves for the herpesviruses quantitative real time PCR

|  | OLIGONUCLEOTIDE SEQUENCES OF SYNTHETIC CURVE | Reference |
| --- | --- | --- |
|  | **HSV-1**  5'TTCGTGATGTTTGTCACCGCAACGAACGTATGACGTGGTG  CGCGGGATGCGTTCGATGATCATGGTGCAGTTGGCTGATA-3’ | **Lima *et al*., 2017** |
|  | **HSV-2**  5'ATGCTATCTACCCACAACAGACCCACGTACGTACGATCTGG  TACTCGAATGTCTCCGCGCATGCAGGGAAGCATTTACGAGAGCG  CTGATC-3' | **Lima *et al.,* 2017** |
|  | **VZV**  5'CGATATCGCGTGCTGCGGCGCTATGGCGTCGTGTGTTTCCA  TTGCTGAATCGTGCATAGACGGGCCATGCCGTACTG-3' | **this study** |
|  | **CMV**  5'-TTCGTGGCCTCGTAGTGAAAATTAATGGTCGTATTTGAACAGA  TCGCGCACCAATACGGATGCGTTCCTGCAGACAGTAACGGCCCT  GATA-3' | **Raposo *et al*., 2019** |
|  | **HHV-6**  5'-TTCGTGCAAGCTCATGAACATCGTCACGTATACCGATCCCAGC  TCACCACCATCTAAATGCGTAGGTAGCGGCAATTTAGGTCTTTCTGATA-3' | **Raposo *et al*., 2019** |
|  | **HHV-7**  5'-TTCGTCCAATCCTTCCGAAACCGATCGTATCATGGCCAACAAG  CAATCTGCGAGATGCGTTTGTCATTACTCCAGTGACTTCCGCTGATA-3' | **Raposo *et al.,*2019** |
|  | **EBV**  5´TTCGTGGAAACCAGGGAGGCAAATCCGTATCCATCGTCAAAGC  TGCAATGCGTTGATATTGCAGGTAGGAGCGGCTGATA 3’ | **this study** |
|  | **HHV8 ORF73**  5´TTCGTGGAACGCGCCTCATACGACGTATCTCCAGGTCTGTG  GGGTGGTGATGTATGCGTTGAGTACATAGCGGTATTCGCGCTG  ATA 3’ | **this study** |

**Supplement Table S2**

Association between TTV, herpesviruses and cytokines in plasma of women with twin pregnancies

| Cytokine | Trimester | Median (IQR) herpes positive | Median (IQR)  TTV positive | Median (IQR) herpes negative | Median (IQR) TTV negative |
| --- | --- | --- | --- | --- | --- |
| IL-1β | 1^st^ | 2.0 (0.7, 4.8) | 2.3 (0.7, 5.8) | 3.2 (1.7, 5.9) | 3.8 (2.9, 4.7) |
|  | 2^nd^ | 5.4 (3.0, 9.2) | 5.8 (4.5, 8.8) | 5.5 (4.0, 8.6) | 7.3 (3.5, 10.3) |
|  | 3^rd^ | 7.5 (4.2, 13.2) | 10.5 (7.1, 12.5) | 7.9 (5.5, 11.6) | 9.7(5.1, 12.7) |
| IL-6 | 1^st^ | 0.30 (0.18, 0.39) | 0.30 (0.20, 0.31) | 0.30 (0.22, 0.48) | 0.26 (0.21, 0.38) |
|  | 2^nd^ | 0.30 (0.21, 0.76) | 0.30 (0.28, 0.56) | 0.31 (0.23, 0.63) | 0.35 (0.25, 0.68) |
|  | 3^rd^ | 0.62 (0.30, 1.3) | 0.51 (0.30, 0.97) | 0.60 (0.30, 1.1) | 0.66 (0.35, 0.95) |
| TNF-α | 1^st^ | 5.7 (4.4, 9.6) | 7.1 (4.9, 11.3) | 7.3 (5.0, 11.5) | 6.0 (4.2, 9.0) |
|  | 2^nd^ | 7.8 (5.8, 14.9) | 8.2 (5.5, 11.4) | 7.5 (5.0, 11.4) | 7.8 (5.0, 11.4) |
|  | 3^rd^ | 10.1 (8.0, 15.3) | 10.8 (7.4, 17.2) | 10.1 (6.8, 15.8) | 10.1 (9.0, 12.1) |

IQR, interquartile range

**Figure S1.** Viral load of DNA viruses detection in all trimesters.


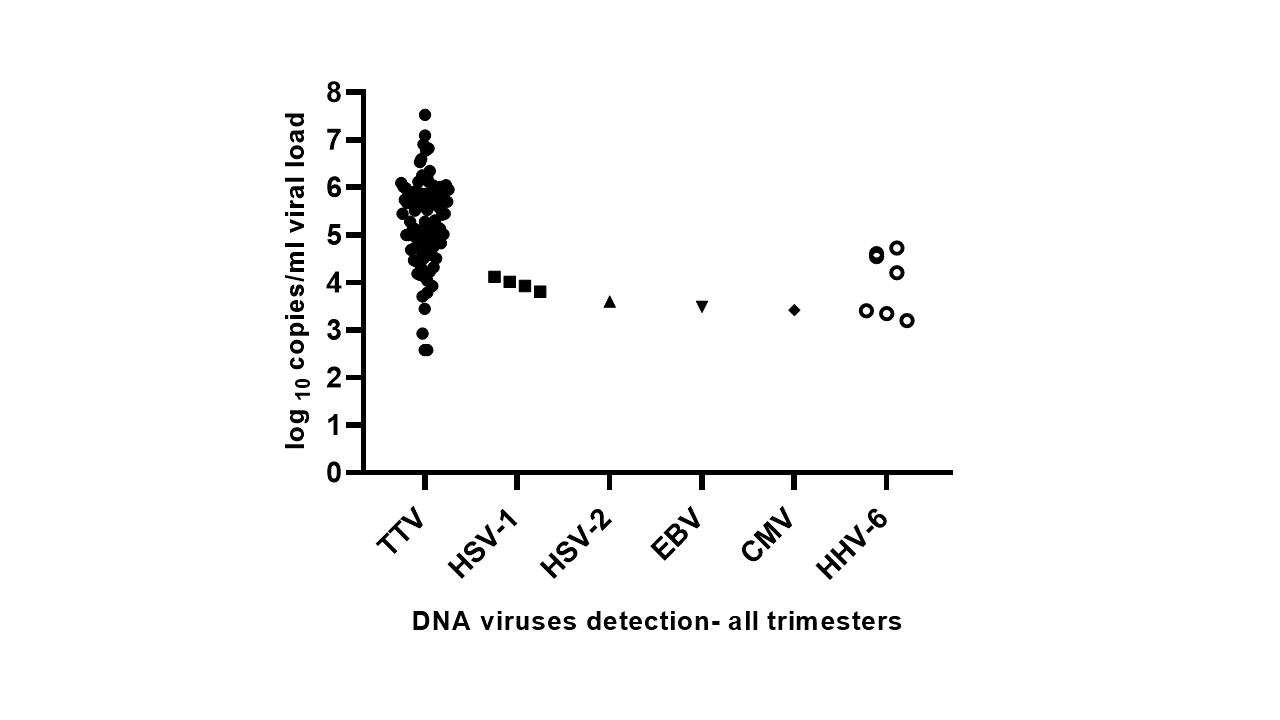

Supplement: S1 File — This file contains all supplementary figures and tables. (DOCX) [file pone.0335200.s001.docx]
